# Structural and Serological Characterization of the O Antigen of *Proteus mirabilis* Clinical Isolates Classified into a New *Proteus* Serogroup, O84

**DOI:** 10.3390/ijms24054699

**Published:** 2023-02-28

**Authors:** Dominika Drzewiecka, Małgorzata Siwińska, Sof’ya N. Senchenkova, Evgeniya A. Levina, Alexander S. Shashkov, Yuriy A. Knirel

**Affiliations:** 1 Department of Biology of Bacteria, Faculty of Biology and Environmental Protection, University of Lodz, 90-237 Lodz, Poland; 2 N. D. Zelinsky Institute of Organic Chemistry, Russian Academy of Sciences, 119991 Moscow, Russia

**Keywords:** *Proteus mirabilis*, lipopolysaccharide (LPS), O serotype, serological classification, Dienes test, O antigen, O-specific polysaccharide (OPS), chemical structure

## Abstract

Two closely related *Proteus mirabilis* smooth strains, Kr1 and Ks20, were isolated from wound and skin samples, respectively, of two infected patients in central Poland. Serological tests, using the rabbit Kr1-specific antiserum, revealed that both strains presented the same O serotype. Their O antigens are unique among the *Proteus* O serotypes, which had been described earlier, as they were not recognized in an enzyme-linked immunosorbent assay (ELISA) by a set of *Proteus* O1-O83 antisera. Additionally, the Kr1 antiserum did not react with O1-O83 lipopolysaccharides (LPSs). The O-specific polysaccharide (OPS, O antigen) of *P. mirabilis* Kr1 was obtained via the mild acid degradation of the LPSs, and its structure was established via a chemical analysis and one- and two-dimensional ^1^H and ^13^C nuclear magnetic resonance (NMR) spectroscopy applied to both initial and O-deacetylated polysaccharides, where most β-2-acetamido-2-deoxyglucose (N-acetylglucosamine) (GlcNAc) residues are non-stoichiometrically O-acetylated at positions 3, 4, and 6 or 3 and 6, and a minority of α-GlcNAc residues are 6-O-acetylated. Based on the serological features and chemical data, *P. mirabilis* Kr1 and Ks20 were proposed as candidates to a new successive O-serogroup in the genus *Proteus*, O84, which is another example of new *Proteus* O serotypes identified lately among serologically differentiated *Proteus* bacilli infecting patients in central Poland.

## 1. Introduction

Lipopolysaccharides (LPSs) are important cell wall constituents of Gram-negative bacteria, including those belonging to the genus *Proteus* [1,2]. The bacilli represent the family *Morganellaceae* in a new order *Enterobacterales*, covering several families of fermenting intestinal rods belonging to the former family *Enterobacteriaceae* [3]. Although many new species have been described in the genus recently [4], *Proteus mirabilis* is still the species isolated with the highest frequency, especially from clinical materials and also from environmental samples (soil, water, sewage) [1,5,6,7]. The species may occur in the human intestines and fecally contaminated water [5,6,8]. *P. mirabilis* is an opportunistic pathogen, which may cause complicated urinary tract infections, most often in people with an impaired immune system and long-term catheterized patients [8,9,10]. It is considered to be one of the most common pathogens causing urinary tract infections [2,8,11,12] and is also a frequent etiological factor of wound infections [6,11,13,14,15,16]. It has been isolated from feces, blood, and pus [1,5,10]. The bacteria manage to overcome the immunity barriers of a host organism due to many virulence factors, which they produce [2,17]. One of them is the ability to swarm across wet solid surfaces, which is a very typical feature of *Proteus* spp. bacteria, and strongly expressed by *P. mirabilis* [18,19]. It may be employed to study a mutual relatedness between different isolates using the Dienes test [5,8,18]. This simple and reliable method enables the differentiation of non-kin strains as they form a visible Dienes line between their swarms, recognizing the opponent strain as non-self. On the other hand, the related strains do not form a borderline and their swarms mix with each other [5,8,18,20]. The swarming phenomenon involving cyclic morphological changes of bacterial cells is presented by the smooth (S) forms only, producing full-length LPS molecules, which are another important pathogenic factor of the bacteria [2,21].

The fine chemical structure of the O-specific polysaccharides (OPS) of the LPSs determines the serospecificity of the bacteria [2,22]. Currently, the serological classification scheme of *Proteus* spp. covers as many as 83 O serogroups [23], reflecting a large serological variety among clinical strains representing this genus [16,22]. The scheme is being constantly updated and broadened as new *Proteus* O serotypes are still being discovered in patients, which are characterized by a diverse and unique structure of the O-antigen repeating unit in their LPSs. However, some of the serogroups have been observed to be predominant in patients for many years [24].

Recently, we have studied the serological diversity of clinical strains of *Proteus* spp. isolated in central Poland and several new O serotypes have been described, including the O78 serogroup, which is prevalent in this area [16]. Here, we studied the O antigens of two more *P. mirabilis* strains Kr1 and Ks20, which were isolated from a wound and skin, respectively, of two Polish patients. These isolates are representatives of a new serogroup, O84, proposed to be created within the genus *Proteus*.

## 2. Results and Discussion

### 2.1. Characterization of Strains

The studied strains Kr1 and Ks20 were recognized as belonging to the *P. mirabilis* species, which was confirmed on the basis of their metabolic features presented when they were inoculated on the selected media proposed by Senior [25]. Both strains expressed phenylalanine deamination, urease production, and ornithine decarboxylation; however, they were not able to ferment mannose or salicine and could not produce indole from tryptophane.

The thermostability and pseudoagglutination in 0.85% NaCl tests enabled the determination of the S phenotype of both isolates. The strains formed a homogenous suspension in the NaCl solution, which remained stable in the medium during the boiling process. They were also characterized by the capability to effectively swarm at distances of 25–26 cm from the place of inoculation. Such an ability is a typical feature of *P. mirabilis* strains, as more than 90% of *P. mirabilis* strains studied before, including the currently described ones, were capable of moving on a solid surface, and as much as 70% presented intensive swarming growth [16].

**Figure 1:**
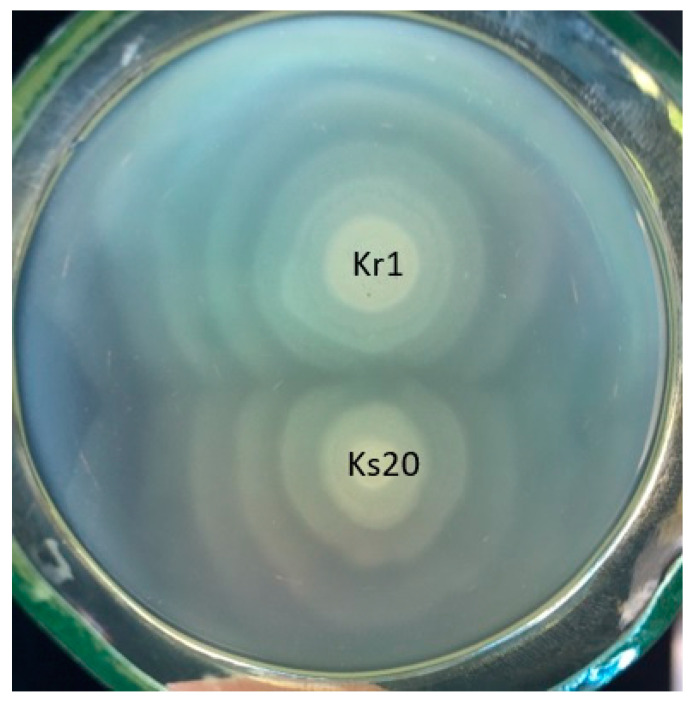
The Dienes test for the studied *P. mirabilis* Kr1 and Ks20 isolates on nutrient agar plates. The swarms of the strains have merged and no line of demarcation is visible between them.

The swarming ability of both isolates gave the opportunity to check their mutual relatedness by employing the Dienes test. This is a simple but reliable method (comparable to ribotyping) used to analyze a kinship of swarming strains [26,27,28]. It may be successfully used to prove the relatedness of *P. mirabilis* [29,30] or *Pseudomonas aeruginosa* [31] isolates capable of moving over the solid media. As a result, we observed no demarcation line between the swarms of the studied strains, which indicated that they are closely related (Figure 1). Therefore, the strains could have been transmitted between the two patients as a result of a nosocomial infection [32,33].

Thus, both strains were recognized as S forms, synthetizing a full three-part LPS.

### 2.2. Serological Studies

In the preliminary step, wet biomasses of the studied strains were tested in an enzyme-linked immunosorbent assay (ELISA) with a set of rabbit antisera specific to all the described O serotypes in the genus *Proteus*. However, no strong cross-reactions comparable to the ones typical of homologous systems were noticed. Thus, both closely related strains, Kr1 and Ks20, are serologically different from the 83 O serotypes described so far in the genus *Proteus*. The Kr1 isolate was selected for further detailed serological investigation. As the epitopes on the OPS might be masked by proteins and other cell elements, the LPS of the Kr1 isolate was extracted from its dry biomass using the phenol–water procedure [34]. It was used as an antigen in the ELISA. Again, no cross-reactions of the O1-O83 rabbit antisera with the LPS were observed.

In the next step, the rabbit O antiserum against the Kr1 strain was obtained and its reactivity in the homologous system (with the Kr1 LPS) in the ELISA gained the titer range of 1:128,000–1:256,000. A similar reaction of the antiserum with the biomass of the other studied strain, Ks20, was observed (Table 1). However, no cross-reactions were observed when the Kr1 antiserum was studied in an ELISA with the set of O1-O83 LPSs. These results confirmed that the isolates studied are serologically unique.

**Figure 2:**
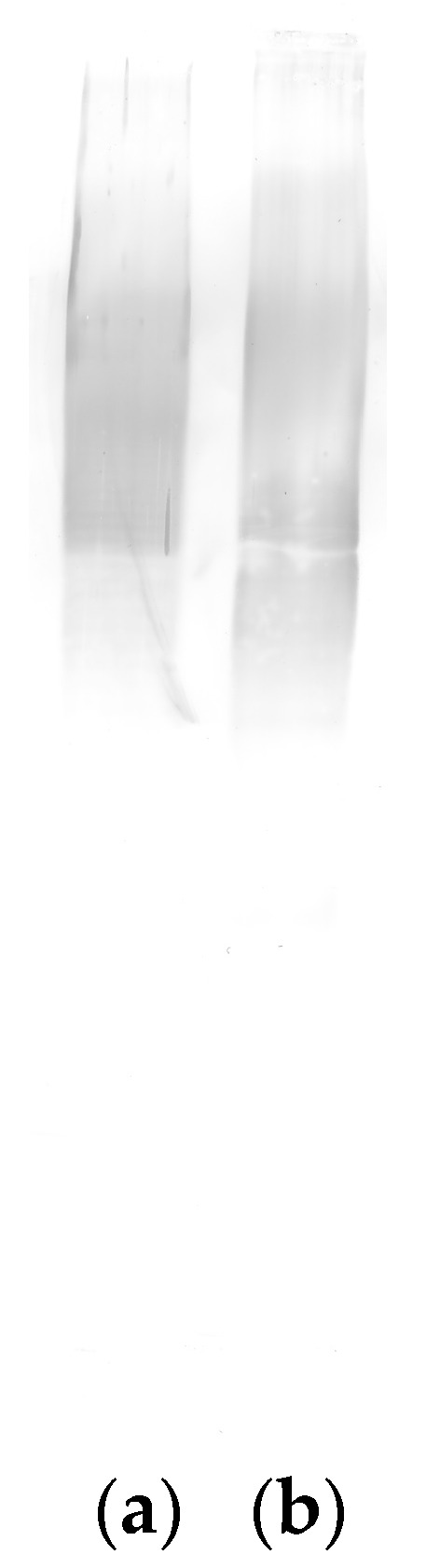
Western blotting of *P. mirabilis* Kr1 LPS (**a**) and *P. mirabilis* Ks20 biomass (**b**) with *P. mirabilis* Kr1 antiserum.

The mutual serological similarity of strains Kr1 and Ks20 was confirmed by Western blotting, which showed similar strong reactions of the Kr1 antiserum with both analyzed strains (Figure 2). Namely, both LPSs presented convergent patterns of reaction with the Kr1 antiserum when separated in SDS-PAGE and transferred into a nitrocellulose membrane. The antiserum recognized specific epitopes in long-chain LPS molecules containing the OPS, which is built up of different numbers of O-repeating units, slowly migrating in the gel. The fast-migrating LPS fractions deprived of the OPS chains were not recognized by the serum (no visible reaction), and hence anti-core antibodies were absent from the serum.

Next, the Kr1 antiserum was adsorbed using the strain Ks20 biomass. The more similar the O antigens are, the more antibodies should be removed by the cross-reacting biomass. Indeed, the reaction of the adsorbed antiserum was totally abolished, both in the ELISA (Table 1) and Western blotting results (no visible patterns), not only with the adsorbing Ks20 strain (the control of the adsorption process) but also in the homologous system (with the Kr1 strain). The results confirmed the serological identity of the O antigens of both strains, which seem to be unique among all *Proteus* O serotypes described so far.

The following chemical studies of the *P. mirabilis* Kr1 OPS revealed the structure of this new-type O antigen.

### 2.3. Structural Studies

A long-chain OPS was obtained via the mild acid degradation of the LPS of *P. mirabilis* Kr1 followed by gel-permeation chromatography on a Sephadex G-50 Superfine instrument. The sugar analysis via gas–liquid chromatography of the acetylated alditols derived from the OPS by acid hydrolysis followed by borohydride reduction and acetylation [35] revealed 2-acetamido-2-deoxyglucose (N-acetylglucosamine) (GlcNAc) and a trace amount of glucose. The gas–liquid chromatography analysis of the acetylated methyl glycosides derived after methanolysis of the OPS and acetylation showed the presence of glucuronic acid (GlcA). Both GlcNAc and GlcA were assumed to have the d configuration, as in all other bacterial polysaccharides studied so far.

The ^1^H and ^13^C nuclear magnetic resonance (NMR) spectra of the OPS showed a heterogeneity, most likely owing to non-stoichiometric O-acetylation (in the NMR spectra, there were signals for multiple O-acetyl groups at δ 2.08–2.15 and 21.5–21.8 (Me)). Indeed, the treatment of the OPS with aqueous ammonia afforded an O-deacetylated polysaccharide (DPS) with a regular structure.

**Figure 3:**
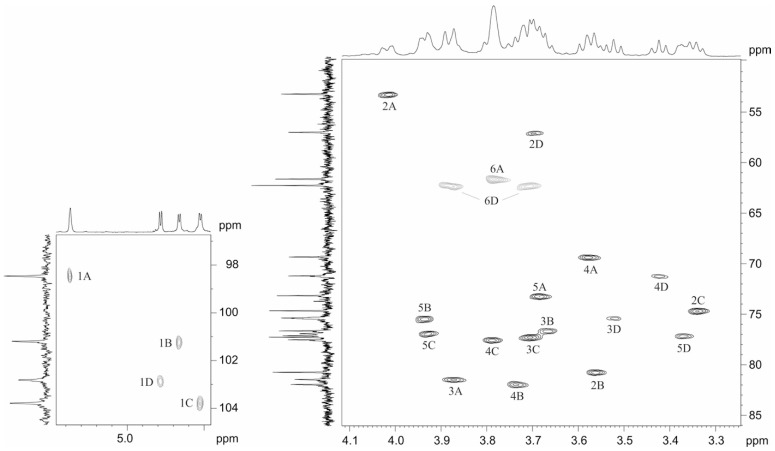
Parts of a two-dimensional ^1^H,^13^C heteronuclear single-quantum correlation (HSQC) spectrum of the O-deacetylated polysaccharide from *P. mirabilis* Kr1. The corresponding parts of the ^1^H and ^13^C nuclear magnetic resonance (NMR) spectra are shown along the horizontal and vertical axes, respectively. The numbers refer to H/C pairs in sugar residues denoted by letters, as indicated in Table 2.

The ^13^C NMR spectrum of the DPS (Figure 3) showed signals for four anomeric carbons at δ 98.5–103.8, two HO*C*H_2_-C groups (C-6 of GlcNAc) at δ 61.6 and 62.3, two nitrogen-bearing carbons (C-2 of GlcNAc) at δ 53.2 and 57.0, non-anomeric sugar-ring oxygen-linked carbons at δ 69.4–82.0, and two carboxyl groups at δ 174.6 and 175.0, as well as two N-acetyl groups at δ 23.5, 23.6 (both CH_3_), and 175.9 (2 CO) (Table 2).

Accordingly, the ^1^H NMR spectrum of the DPS (Figure 3) contained signals for four anomeric protons at δ 4.52–5.37, other sugar protons at δ 3.34–4.02, and two N-acetyl groups at δ 2.04 and 2.06 (Table 2).

The NMR spectra of the DPS were assigned using ^1^H,^1^H correlation spectroscopy (COSY), ^1^H,^1^H total correlation spectroscopy (TOCSY), and ^1^H,^13^C heteronuclear single-quantum correlation (HSQC) (Figure 3) experiments, and spin systems for two residues of β-Glc*p*NAc (units **A** and **D**) and two residues of GlcA (unit **B** and **C**) were identified (Table 2). The two-dimensional ^1^H,^1^H rotating-frame nuclear Overhauser effect correlation spectroscopy (ROESY) spectrum of the DPS showed the following correlations between the anomeric protons and protons at the linkage carbons: **A** H-1/**C** H-4, **C** H-1/**B** H-4, **B** H-1/**A** H-3, and **D** H-1/**B** H-2 at δ 5.37/3.79, 4.52/3.74, 4.66/3.87, and 4.78/3.56, respectively. In the ^1^H,^13^C heteronuclear multiple bond correlation spectroscopy (HMBC) spectrum of the DPS, there were cross-peaks at 5.37/77.6, 4.52/82.0 4.66/81.5, and 4.78/80.8 for correlations between the anomeric protons and linkage carbons **A** H-1/**C**C-4, **C** H-1/**B** C-43, **B** H-1/**A** C-3, and **D** H-1/**B** C-2, respectively.

These data defined the linkages and the sequence of the monosaccharides in the repeating unit of the OPS. The positions of glycosylation of the monosaccharides were confirmed by low-field positions of the signals for the linkage carbons (Table 2), as compared with their positions in the spectra of the corresponding non-substituted monosaccharides [36,37].

**Scheme 1:**
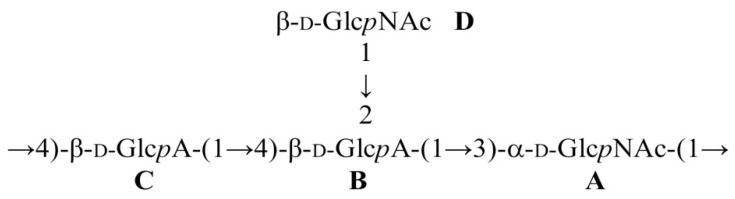
Structure of O-deacetylated polysaccharide (DPS) from *P. mirabilis* Kr1.

Therefore, the DPS from *P. mirabilis* Kr1 has the following structure (Scheme 1).

The comparison of the ^1^H,^13^C HSQC spectra of the DPS and OPS revealed significant displacements of parts of H/C cross-peaks for units **A** and **D**. In particular, parts of the cross-peaks for the **A**6, **D**3, and **D**4 H/C pairs shifted downfield from δ 3.78/61.6, 3.52/75.4, and 3.42/71.2 in the spectrum of the DPS, respectively, to δ 4.19, 4.24/64.1, 4 98/77.0, and 4.83/72.6 in the spectrum of the OPS. Therefore, units **A** and **D** were non-stoichiometrically O-acetylated at the corresponding positions. In addition, two sets of low-field cross-peaks for the H-6/C-6 pairs of unit **D** were observed at 4.24 and 4.45/65.0 and 4.27 and 4.45/64.9 in the spectrum of the DPS, which were assigned to the 6-O-acetylated and 3,6-di-O-acetylated residues, respectively. Based on the ratios of the cross-peak squares, the degrees of O-acetylation of position 6 of unit **A** and positions 3, 4, and 6, and 3 and 6 of unit **D** were estimated as 20, 10, 20, 30, and 30%, respectively.

**Scheme 2:**
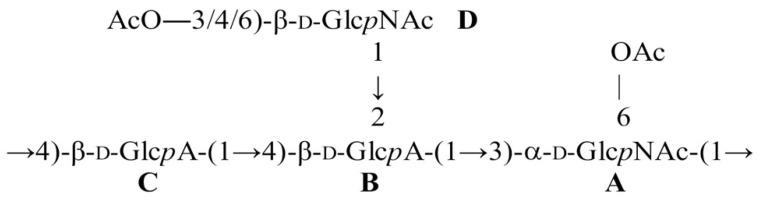
Structure of the O-specific polysaccharide from *P. mirabilis* Kr1. The degree of O-acetylation of α-d-N-acetylglucosamine (Glc*p*NAc) (**A**) at position 6 is 20%; the degrees of O-acetylation of β-d-Glc*p*NAc (**D**) at positions 3, 4, and 6 and 3 and 6 are 10, 20, 30, and 30%, respectively.

Therefore, the OPS from *P. mirabilis* Kr1 has the structure shown in Scheme 2, which is unique among isolates of this species. According to our data, the OPS of *P. mirabilis* Ks20 has the same structure.

### 2.4. Classification of P. mirabilis Kr1 and Ks20

Based on serological and structural data, the two clinical isolates, *P. mirabilis* Kr1 and Ks20, could not be classified into any existing *Proteus* O serogroup and were classified into a new *Proteus* O serogroup, O84. Being closely related and producing the same unique type of O antigen, Kr1 and Ks20 may be one clone transmitted between two patients as a result of a nosocomial infection. Strains of the O84 serogroup are not widespread among patients in central Poland, as only two from six hundred of the clinical isolates studied in the Łódź region, Poland [16], represent this serogroup so far. However, it is not clear how widely they are distributed worldwide. Earlier, representatives of some other *P. mirabilis* O serogroups were recognized as being more widespread among Polish patients in the Łódź region [16]. Many of these prevailing serogroups were also indicated by Larsson in 1984 [24] as dominating in many other countries. The strains belonging to these serogroups seem to cause infections more successfully than other isolates, thereby posing a real threat to patients. However, there are several examples of newly recognized O serotypes in the Łódź area, which are not so common in patients [38,39,40]. There may be two reasons that should be taken into consideration. Firstly, the strains representing the rare serotypes may be much less virulent and less easily transmitted among patients. Secondly, the high serological heterogeneity recognized in the genus *Proteus* may reflect the large potential of the bacteria in terms of their phenotypic (antigenic) variability, e.g., in response to changing environmental conditions or to avoid the reaction of the immune system [8,41].

## 3. Materials and Methods

### 3.1. Bacterial Strains, Identification, Characterization, Cultivation

*P. mirabilis* Kr1 and Ks20 clinical strains were isolated in December 2008 from wound and skin samples, respectively, of a 51-year-old female (patient in the Internal Disease Division) (Kr1) and a 13-year-old girl (patient in the Pediatric Division) (Ks20) in the Biegański Hospital in Łódź, Poland, and were kindly provided by the hospital laboratory. The strains were stored on Luria broth (LB) cultures with 25% glycerol at −80 °C. The isolates were confirmed as representing *P. mirabilis* species by checking their biochemical activities (phenylalanine deamination, ornithine decarboxylation, urease production, tryptophane decomposition to indol, lack of mannose and salicin fermentation abilities) [25].

The S form of the isolates was confirmed, as described [42] based on the lack of spontaneous agglutination of the strains’ biomass in 0.85% NaCl (homogeneous suspension on a slide), the stability of the cells suspended in boiled broth cultures for 2.5 h, and the capability for swarming growth on the surface of the LB 1.5% agar (the radius of the growth zone was measured after 24 h of incubation of spot-inoculated liquid cultures at 37 °C).

The relatedness of the tested isolates was indicated using the Dienes test [26,30]. After 24 h of incubation of the cultures at 37 °C, which had been spot-inoculated on nutrient agar plates, a demarcation line formation was checked on the border of the two strains swarms.

The bacterial masses of both strains were obtained by centrifugation of 18 h aerated liquid broth cultures (BTL, Poland) with the addition of 0.2% glucose, incubated at 37 °C and used as antigens in the serological tests, for LPS extraction and antisera adsorption.

### 3.2. Lipopolysaccharides (LPSs), Antisera

LPS was extracted from the dry (lyophilized) biomass of the Kr1 strain, using the phenol–water procedure [34]. Briefly, the extraction was carried out for 5 min at 67 °C in aqueous 45% phenol added to the biomass at a ratio of 500 mL to 20 g. Then, the mixture was cooled, the residual biomass was removed, and phenol was removed by dialysis firstly against the tap water and then against the distilled water. The LPS was purified by the treatment with aqueous 50% CCl_3_CO_2_H at 4 °C followed by a dialysis of the supernatant against the distilled water [43].

The *P. mirabilis* Kr1 O-specific serum was prepared via the intravenous immunization of a New Zealand white rabbit with a heat-inactivated (100 °C, 2.5 h) bacterial suspension (1.5 × 10^10^ cells per ml) in three doses of 0.25, 0.5, and 1.0 mL, as described earlier [44]. The 18-day antiserum was prepared from the blood taken from the rabbit 8 days after the last injection. All procedures were approved by the local ethical committee.

The *Proteus* O1-O83 LPSs and specific rabbit polyclonal O antisera came from the Department of Biology of Bacteria, University of Łódź, Poland.

The adsorbed O antisera used in the research were prepared by mixing a wet bacterial mass with 0.5 mL of a serum diluted to 1:100 in phosphate-buffered saline (PBS) at a volume ratio of 1:10, with incubation on ice for 30 min. The biomass was then removed by centrifugation and the process was repeated twice [29]. All sera (native and adsorbed) were kept at −20 °C.

### 3.3. Serological Assays

The ELISA as well as the sodium dodecyl sulfate–polyacrylamide gel electrophoresis (SDS-PAGE) and Western blotting tests were performed as previously described [44].

In the ELISA, we used flat-bottom Nunc-Immuno plates that were coated with antigens, namely bacterial masses at a concentration of 5 µg per well or a plate or LPS preparations at a concentration of 50 ng per well, diluted in PBS. Appropriate O antisera were serially diluted on the plates and were the source of specific antibodies. The antibodies bound with antigens were detected by rabbit-IgG-specific peroxidase-conjugated goat antibodies (Jackson ImmunoResearch, West Grove, PA, USA). After the addition of the peroxidase substrate 2,2’-Azino-bis(3-ethylbenzothiazoline-6-sulfonic acid) diammonium salt (ABTS) (Sigma-Aldrich, St. Louis, MI, USA) with 0.1% H_2_O_2_, the last antiserum dilution with the final absorbance of (A_405_) ≥0.2, read with the use of a Multiskan Go microplate reader (Thermo Fisher Scientific, Waltham, MA, USA), was considered as a titer.

The samples for the SDS-PAGE test were prepared as follows: bacterial masses (10 mg/1 mL of distilled water) were mixed with a sample buffer at a volume ratio 1:1 and proteinase K (10 mg/mL) (at a volume ratio of 1:8) and incubated for 1 h at 60 °C; LPSs (2 mg/mL of distilled water) were suspended in a sample buffer (1:1) and boiled for 10 min before being applied to the gel. The separated samples were transferred to nitrocellulose. The reaction with a specific O-antiserum was visualized via Western blotting using goat anti-rabbit-IgG antibodies conjugated with alkaline phosphatase (AP) (Jackson ImmunoResearch, West Grove, PA, USA) and a proper AP Conjugate Substrate Kit (Bio-Rad, Hercules, CA, USA).

### 3.4. Isolation of O-Specific Polysaccharide Samples

An LPS sample was hydrolyzed with aqueous 2% HOAc (4 mL) at 100 °C for 2 h, a lipid precipitate was removed by centrifugation (13,000× *g*, 20 min), and an OPS sample was obtained by fractionation of the supernatant by GPC on a column (60 × 2.5 cm) of Sephadex G-50 Superfine (Amersham Biosciences, Uppsala, Sweden) in 0.05 M pyridinium acetate buffer at pH 4.5, monitored with a differential refractometer (Knauer, Germany).

### 3.5. O-Deacetylation

An OPS sample was heated with 12.5% aqueous ammonia (2 mL) at 37 °C for 16 h, then the ammonia was removed with a stream of air and the remaining solution was freeze-dried to give a DPS sample.

### 3.6. Composition Analysis

For the neutral and amino sugar analyses, an OPS sample (0.5 mg) was subjected to acid hydrolysis (2 M CF_3_CO_2_H, 120 °C, 2 h) followed by reduction with an excess of NaBH_4_ (20 °C, 2 h), acetylation with a 1:1 (*v*/*v*) Ac_2_O–pyridine mixture (20 °C, 16 h), and an analysis via GLC on an Agilent 7820A GC System (Maestro, Moscow, Russia) equipped with an HP-5ms column (Agilent) using a temperature gradient of 7 °C min^−1^ from 160 to 290 °C. For the uronic acid analysis, an OPS sample (0.5 mg) was subjected to methanolysis (1 M HCl in MeOH, 85 °C, 24 h) followed by acetylation as described above.

### 3.7. Nuclear Magnetic Resonance (NMR) Spectroscopy

The NMR spectra were recorded in 99.95% D_2_O (after deuterium-exchange by freeze-drying from 99.9% D_2_O) at 20 °C on a Bruker Avance II 600 MHz spectrometer. The Bruker TopSpin 2.1 program was used to acquire and process the NMR data. Internal sodium 3-trimethylsilylpropanoate-2,2,3,3-d_4_ (δ_H_ 0, δ_C_ −1.6) was used as a reference for calibration. Mixing times of 200 and 150 ms were used in the two-dimensional TOCSY and ROESY experiments, respectively.

## Figures and Tables

**Table 1:** The reactivity of intact and adsorbed *P. mirabilis* Kr1 O antiserum samples with the studied antigens in enzyme-linked immunosorbent assay (ELISA).

*P. mirabilis* Kr1 O Antiserum	Reciprocal Titer in the Reaction with	
	Kr1 LPS	Ks20 Biomass
not adsorbed	128,000	256,000
adsorbed with *P. mirabilis* Ks20 biomass	<1000	<1000

**Table 2:** ^1^H and ^13^C nuclear magnetic resonance (NMR) chemical shifts (δ, ppm) of the O-deacetylated polysaccharide (DPS) from *P. mirabilis* Kr1.

Monosaccharide Residue	C-1 *H-1*	C-2 *H-2*	C-3 *H-3*	C-4 *H-4*	C-5 *H-5*	C-6 *H-6 (6a, 6b)*
→3)-α-d-Glc*p*NAc-(1→ **A**	98.5	53.2	81.5	69.4	73.3	61.6
	*5.37*	*4.02*	*3.87*	*3.58*	*3.68*	*3.78, 3.78*
→2,4)-β-d-Glc*p*A-(1→ **B**	101.3	80.8	76.7	82.0	75.5	174.5 ^a^
	*4.66*	*3.56*	*3.67*	*3.74*	*3.94*	
→4)-β-d-Glc*p*A-(1→ **C**	103.8	74.7	77.4	77.6	77.0	174.4 ^a^
	*4.52*	*3.34*	*3.71*	*3.79*	*3.93*	
β-d-Glc*p*NAc-(1→ **D**	102.9	57.0	75.4	71.2	77.2	62.3
	*4.78*	*3.70*	*3.52*	*3.42*	*3.37*	*3.71, 3.89*

^1^H NMR chemical shifts are italicized. Chemical shifts for the N-acetyl groups are δ_C_ 23.5, 23.6 (both CH_3_), 174.6, and 175.0 (both CO); δ_H_ 2.04 and 2.06. ^a^ The assignment could be interchanged.

## Data Availability

The data that support the findings of this study are available from the corresponding author on reasonable request.
